# Contribution of T- and B-cell intrinsic toll-like receptors to the adaptive immune response in viral infectious diseases

**DOI:** 10.1007/s00018-022-04582-x

**Published:** 2022-10-12

**Authors:** Ejuan Zhang, Zhiyong Ma, Mengji Lu

**Affiliations:** 1 Medical Science Research Center, Zhongnan Hospital of Wuhan University, Wuhan, China; 2 Department of Infectious Diseases, Zhongnan Hospital of Wuhan University, Wuhan, China; 3 Institute for Virology, University Hospital of Essen, University of Duisburg-Essen, Hufelandstr. 55, 45122 Essen, Germany

**Keywords:** Toll-like receptor, Adaptive immune response, T cells, B cells, Viral infection, Immunotherapy

## Abstract

Toll-like receptors (TLRs) comprise a class of highly conserved molecules that recognize pathogen-associated molecular patterns and play a vital role in host defense against multiple viral infectious diseases. Although TLRs are highly expressed on innate immune cells and play indirect roles in regulating antiviral adaptive immune responses, intrinsic expression of TLRs in adaptive immune cells, including T cells and B cells, cannot be ignored. TLRs expressed in CD4 + and CD8 + T cells play roles in enhancing TCR signal-induced T-cell activation, proliferation, function, and survival, serving as costimulatory molecules. Gene knockout of TLR signaling molecules has been shown to diminish antiviral adaptive immune responses and affect viral clearance in multiple viral infectious animal models. These results have highlighted the critical role of TLRs in the long-term immunological control of viral infection. This review summarizes the expression and function of TLR signaling pathways in T and B cells, focusing on the in vitro and vivo mechanisms and effects of intrinsic TLR signaling in regulating T- and B-cell responses during viral infection. The potential clinical use of TLR-based immune regulatory drugs for viral infectious diseases is also explored.

## Introduction

Adaptive immune responses, including antigen-specific antibodies and CD8 + T cells, play a critical role in controlling viral infections. Antibodies against viral proteins inhibit viral infection by neutralizing viral particles or mediating the killing of infected cells through antibody-dependent cell-mediated cytotoxicity (ADCC) [1, 2]. Viral-specific CD8 + T cells contribute to viral control by both cytolytic destruction of infected cells and noncytolytic mechanisms after recognizing the viral-derived peptides presented by major histocompatibility complexes (MHCs) [3, 4]. The essential role of humoral and cellular immune responses for viral clearance during acute hepatitis B virus (HBV) and hepatitis C virus (HCV) infection, as well as other viral infectious diseases, has been well documented. In contrast, deficiency or exhaustion of viral-specific B and T-cell responses often leads to viral persistence [5–7].

Rapid and immediate surveillance of viral infections is achieved by the innate immune system through the detection of pathogen-associated molecular patterns (PAMPs) by host pattern-recognition receptors (PRRs), such as Toll-like receptors (TLRs), RIG-I-like receptors (RLRs), NOD-like receptors (NLRs), and C-type lectin receptors (CLRs). TLRs comprise a class of highly conserved molecules that play a vital role in host defense against many pathogenic microorganisms [8]. TLRs are widely expressed in a broad range of tissues and cell types. Activation of TLR signaling pathways by PAMP recognition suppresses the replication and spread of invading pathogens by rapidly inducing antiviral/antimicrobial molecules such as type I interferon (IFN) and TNF-α, modulating the activation of protective viral-specific adaptive immune responses [9, 10]. The TLR signaling pathway regulates adaptive immune cells through either indirect or direct mechanisms. Activation of TLR signaling pathways in antigen presenting cells (APCs) regulates the activation and maturation of dendritic cells (DCs), differentiation of macrophages, presentation and cross-presentation of antigens, and the production of proinflammatory cytokines and chemokines [11]. In several published studies, DCs stimulated by TLR2, 3, 7, and 9 agonists tend to support Th1/CD8 + T-cell responses, while TLR5 agonists enhance Th2/B-cell responses [12–15]. Importantly, intrinsic expression of TLRs in lymphocytes has been characterized, and their downregulation is apparently associated with chronic viral infections [16, 17]. However, the role of TLR signaling pathways in T and B cells is often overlooked. In recent years, it has become evident using in vitro cell models and knockout mice that activation of intrinsic TLR signaling pathways in T/B cells may also play an essential role in the maturation and maintenance of protective immune responses in tumor and infectious diseases [18, 19]. This review summarizes the underlying mechanisms of the intrinsic TLR signaling pathway in regulating B- and T-cell responses and potential application of the TLR signaling pathway in clinical treatments.

## Expression of TLRs in T and B cells

The IL-1R/TLR superfamily is a group of receptors that are mammalian homologues to the Toll receptors that were originally discovered in Drosophila. To date, 10 TLRs have been described in humans (TLR1-TLR10) and 13 in mice (TLR1-13, including 12 functional TLRs and a disrupted pseudogene TLR10) [20]. TLRs are type I transmembrane glycoproteins located on the cell surface (TLR1, 2, 4, 5, 6, 10) or within endosomes (TLR 3, 7, 8, 9, 11, 12, 13). All TLRs are composed of three principal domains: a leucine-rich N-terminal extracellular domain, a single-spanning transmembrane domain and a conserved C-terminal intracellular toll/IL-1R (TIR) domain. The extracellular domain binds to and recognizes agonists, and the intracellular domain initiates downstream signal cascades by recruiting adaptor proteins such as MyD88 or TRIF, which activate NF-kB, MAPK or IRFs to regulate the production of IFN-I, proinflammatory cytokines and chemokines [21].

Expression of TLRs in T cells has been reported, but the results are variable with respect to the animal model, mouse strain, cell or tissue type and disease progression that was used (Table 1). Generally, CD4 + and CD8 + T cells express functional TLRs. In humans, peripheral CD4 + T cells express almost all TLRs, including TLR1-5, TLR7/8, and TLR9, at the mRNA level, while CD8 + T cells express TLR1/2, TLR3, TLR4, and TLR5 at both the mRNA and protein levels [22–25]. Mouse CD4 + T cells express all TLRs at the mRNA level [26, 27], while TLR mRNA found in murine CD8 + T cells is limited to TLR1/2/6, TLR7 and TLR9 in both naïve and activated cells [28]. Moreover, functional stimulation indicates that CD8 + T cells respond to extracellular TLR2 in the heterodimeric form of TLR1/2 or TLR2/6 and intracellular TLR7 [29]. Expression of those TLRs in CD4 + and CD8 + T cells is related to cell activation, viral infection and IFN stimulation. Naïve CD4 + and CD8 + T cells express relatively low levels of TLRs, while activated or memory CD4 + and CD8 + T cells express most TLRs at significantly increased levels, such as TLR2 and TLR7 [30]. Impaired expression of TLR2 and TLR3 was observed in PBMCs isolated from chronic HBV-infected (CHB) patients at both the mRNA and protein levels [16, 31], while increased expression of TLR1/2, TLR2/6, TLR5, and TLR8 has been reported in CD4 + and CD8 + T cells of CHB-related acute-on-chronic liver failure (ACLF) patients [32]. These variable results may be due to patients enrolled at different stages of disease progression in different studies, which indicates that patients with higher levels of inflammation or liver injury may have increased expression of TLRs on PBMCs. Accordingly, antiviral treatment with IFN-α or nucleoside analogues (NAs) may reverse the abnormal expression of TLR2 and TLR3 in PBMCs of CHB patients, likely by reducing inflammation [31]. These reports indicate broad expression of TLRs in T cells in both humans and mice. Viral infection and antiviral treatment may regulate the expression of TLRs and thus affect the activation of TLR signaling pathways in these cells.

**Table 1:** TLR expression in T and B cells of human and mice

TLR	Human			Mouse		
	CD4	CD8	B cell	CD4	CD8	B cell
1	+ + + +	+ +	+ + + + +	+ +	+ + +	+ + + +
2	+ + +	+ +	+	+ +	+	+ +
3	+ + +	+ +	b.d	+	+	b.d
4	+	+	+	+	b.d	+ +
5	+ + + +	+ + +	b.d	+ +	b.d	b.d
6	+	b.d	+ + + +	+ +	+	+ +
7	+	+	+ +	+	+	+ +
8	+ −	b.d	b.d	+	b.d	b.d
9	+ +	+	+ +	+	+	+ + + +
10	+ +	+	+ + +	n.d	n.d	n.d

Abbreviations: *TLR* toll-like receptor, *b.d* below detection, *n.d* not detected

Expression of TLRs in B cells also varies depending on the B-cell subset and mammalian species (Table 1) [33, 34]. In humans, naïve B cells express low to undetectable levels of TLRs. However, activated and memory B cells exhibit upregulated expression of TLR1, TLR2, TLR6, TLR7, TLR9, and TLR10 after activation via BCR or CD40 stimulation [34–36], and this phenomenon is especially prominent for TLR9 and TLR10 [37]. Murine naïve B cells express a variety of TLRs, including TLR1, TLR2, TLR4, TLR6, TLR7, and TLR9, and they proliferate and secrete antibodies against a variety of TLR agonists in vitro in the absence of BCR cross-linking [38, 39]. Interestingly, unlike human B cells, murine B cells do not express TLR10 but express TLR4 and can be potently activated by LPS [34]. Both human and murine B cells express low levels of TLR3, making them responsive to TLR3 ligands [40]. In addition to upregulation of TLRs by BCR or CD40 stimulation, several studies have demonstrated that cytokines, such as type I interferon, stimulate human, and murine B cells to express TLR3 and TLR7 [41–43]. Differences in the expression pattern between humans and mice may suggest that in the human immune system, TLR-mediated activation of B lymphocytes may be more tightly regulated to avoid an overactivated immune response [33].

## Direct regulation of T-cell activation and function by the intrinsic TLR signaling pathway

### TLRs act as costimulatory molecules to enhance cytokine production

Activation of CD4 + and CD8 + T cells requires TCR signaling that is initiated by recognition of the MHC/peptide complex and is transmitted by the CD3 molecule. The costimulatory signals mediated by the interaction of CD28 or ICOS with their ligands CD80/CD86 or ICOSL, respectively, are indispensable for activating transcription factors, such as nuclear factor-κB (NF-κB), nuclear factor of activated T cells (NFAT), and activator protein 1 (AP1) [44, 45]. Proinflammatory cytokines such as IL-12, IL-4, and IL-17 provide synergistic signaling to induce TFs such as T-bet, GATA-3, and ROR-γτ, which control the differentiation of CD4 + T cells and the downstream production of cytokines [46].

In vitro and in vivo studies have revealed that TLRs expressed in CD4 + and CD8 + T cells play roles in enhancing TCR signal-induced T-cell activation, function, and survival, serving as costimulatory molecules. Several studies found that the costimulatory signals produced by TLR2, TLR3, and TLR9 improve TCR-induced activation of NF-κB and NFAT, which results in amplification of TCR signaling in in vitro differentiated Th1 cells [47, 48]. A recent study of Imanishi et al. further indicated a critical role of the TIRAP-mTORC1 axis in TLR2-mediated IFN-γ production by effector T cells but not naïve cells [49]. CD8 + T cells costimulated by TLR2 or TLR7 agonists require fewer costimulatory signals provided by APCs, lower the threshold of antigen concentrations, and develop into functional memory cells during the antigen-specific activation of TCR-signaling pathway using peptide-presenting APCs [28, 48]. Costimulation of CD8 + T cells by TLR2 or TLR7 agonists significantly improves the expression of T-bet and enhances cell proliferation and cytokine production (IL-2, IFN-γ, and TNF-α) [48, 50]. Activation of TLR2 on CD8 + T cells improves differentiation of the functional memory cell phenotype, characterized by CD127 expression, high levels of CD44 and Ly6C, and low levels of CD122 [51]. The TLR5 agonist flagellin exhibits weaker effects but does indeed participates in improving proliferation and cytokine production in human neonatal CD8 + T cells but not in mouse splenic CD8 + T cells, in agreement with the expression of TLR5 in human and mouse CD8 + T cells [23, 24, 52]. Engagement of TLR agonists with receptors on CD4 + T cells leads to not only the activation and proliferation of cells but also the differentiation of Th1, Th2, and Th17 subtypes [47, 53]. Most studies agree that TLR2 and TLR7 stimulation improves Th1-cell differentiation with increased production of IFN-γ, while several studies reported that TLR2 upregulates Th9 or Th17-cell differentiation [47, 54, 55]. Chodisetti et al. reported that activation of TLR2 during CD4 + T-cell stimulation limits the functional exhaustion induced by long-term stimulation with anti-CD3/CD28 [56]. In contrast to TLR3- or TLR9-stimulated DCs, which improve Th1-cell differentiation, costimulation of TLR3 or TLR9 agonists in cooperation with anti-CD3/CD28 induces Th2 cell differentiation by enhancing expression of the Th2-master transcription factor GATA-3 and suppressing the Th1-master transcription factor T-bet [53, 57].

Interestingly, several groups have reported that some TLRs may directly activate CD4 + and CD8 + T cells in a TCR signaling-independent manner. Due to the relatively low expression of most TLRs in naïve T cells, few studies reported the direct activation of TLR-signaling pathway in naïve T cells. In majority of these reports, purified naïve CD4 + T cells show no significantly changes in cytokine production after TLR agonist stimulation in the absence of TCR stimulation [48, 49]. However, Caron et al. demonstrated that in human CD4 + T cells isolated from healthy volunteers, agonists for TLR2, 5, 7/8 upregulate proliferation and IFN-γ production without costimulation of anti-CD3 or other TCR activators [23]. Specifically, they noticed that isolated CD4^+^CD45RA^+^ naïve T cells responded to the combined stimulation of TLR ligands and IL-2, leading to improved IFN-γ production and cell proliferation despite a relatively lower level than that observed in CD4^+^CD45RO^+^ memory cells. Their results pointed out the potential effect of TLR signal transduction in naïve CD4 + T cells, and thus the roles of TLRs in naïve T-cell activation need to be critically reconsidered. Most studies focused on the pre-activated T cells or memory cells in which the TLR expression was significantly increased. In accordance with the upregulated TLR expression in activated CD4 + T cells, activated or memory CD4 + T cells display much higher sensitivity to TLRs. Other groups using in vitro prepared CD4 + or CD8 + T cells observed similar results after stimulation with TLR agonists. Imanishi et al. found that TLR2 agonist treatment increased the proliferation and IFN-γ production of murine Th1 cells that were differentiated in vitro by anti-CD3 and anti-IL-12 [47]. Rubtsova et al. also found improved IFN-γ production of murine memory CD4 + and CD8 + T cells in response to TLR7 agonist stimulation especially in combination of IL-12 [58]. Gelman et al. reported that TLR3 and TLR9 improved the survival of anti-CD3 pre-activated CD4 + T cells or TCR-transgenic T cells activated by peptide-loaded APCs [26]. Mechanistically, TLR2-mediated bystander activation in Th1 cells is MyD88/IRAK4 dependent, leading to strong and sustained activation of NF-κB and MAPK signals, which are important in controlling T-cell-mediated inflammatory responses [47]. In TLR3- and TLR9-stimulated CD4 + T cells, NF-κB but not MAPK p38 or ERK1/2 activation is required for the survival of activated CD4^+^ T cells [26]. Few studies have reported the direct stimulatory role of TLRs in CD8 + T cells. Studies in murine CD8^+^ T cells have shown that TLR2 and TLR7 agonists stimulate antigen-experienced CD8 + T cells, resulting in rapid production of IFN-γ but not TNF-α or IL-2 [59]. Cytokines such as IL-7 and IL-2 act in synergy with TLR2 to improve the proliferation and IFN-γ production, respectively, of memory CD8 + T cells [60]. Naïve CD8 + T cells theoretically do not respond to singular stimulation by TLR2 agonists. Our unpublished data demonstrated that TLR2-pretreated TCR-transgenic CD8 + T cells exhibit higher levels of CD44 expression and IFN-γ production than nontreated CD8 + T cells after activation by peptide-loaded DCs. It is of interest to further investigate which subtype of CD8 + T cells responds to TLR2 since CD8 + T cells isolated from naïve mice are composed of a heterogeneous reservoir, in which memory cells might respond to TLR2 and act as initiators by producing IFN-γ. No studies have reported the direct stimulatory activity of TLR4 in either human or murine CD4 + or CD8 + T cells.

### TLRs improve the reprogramming of cellular metabolism

Upon viral infection, T cells undergo activation and differentiation processes to develop adaptive antiviral activity, accompanied by reprogramming of cellular metabolism to meet the demands of bioenergy and intermediate substrates for biosynthesis. While naïve T cells primarily obtain energy in the form of ATP from mitochondrial oxidative phosphorylation (OXPHO) and fatty acid oxidation (FAO), activated T cells switch their metabolic program to aerobic glycolysis [61]. Antigen recognition of TCR triggers metabolic reprogramming through several signaling pathways, including PI3K-Akt, MAPK, and mTOR, resulting in a marked increase in glucose and amino acid uptake to improve glycolysis and glutaminolysis [62]. Following the clearance of virus and viral antigens, differentiation of memory cells reverts metabolic reprogramming towards decreased glycolysis and increased OXPHO and FAO, which is dependent on the IL-7, IL-15, and AMPK signaling pathways. During chronic viral infection, mitochondrial dysfunction and reactive oxygen species (ROS) are involved in the functional exhaustion of viral-specific T cells, likely due to the imbalanced utilization of glycolysis and oxidative phosphorylation metabolic pathways in the absence of glucose supply [63]. Upregulated expression of PD-1 in exhausted T cells suppresses TCR signaling and inhibits activation of the PI3K-Akt-mTOR pathway, thus reducing glucose uptake and use and leading to bioenergetic insufficiencies during the early and late stages of infection [64]. Treatment with IL-12, anti-PD-1/PD-L1 and mitochondrial-targeted antioxidants partially reversed the function of exhausted T cells by improving their mitochondrial potential and reducing their dependence on glycolysis [65]. These reports note the central role of cellular metabolism in regulating the activation, function, and fate of viral-specific T cells.

Following TCR signaling-triggered reprogramming of aerobic glycolysis, TLR2 and TLR7 engagement significantly upregulate expression of Glut1, which serves as the key transporter of glucose in T cells and enhances glucose uptake. Meanwhile, a group of important genes for glycolysis are upregulated. Metabolic analysis reveals that both glycolysis and mitochondrial respiration are enhanced upon costimulation of TLR2 and TLR7 [66, 67]. Moreover, glutaminolysis is upregulated by TLR2 and TLR7 costimulation. The TLR2 and TLR7 agonist-induced costimulatory effect is reduced or abolished by chemical blockade of glycolysis or glutaminolysis or removal of glucose or glutamine from the culture medium. Upon TLR2 and TLR7 costimulation, PI3K-Akt-mTOR signaling is required to enhance cytokine production [66]. Blockade of Akt, mTOR or PKC significantly suppresses the costimulatory effects of TLR2 and TLR7, indicating the central role of PI3K-Akt-mTOR in the crosstalk among TCR-signaling, TLR-MyD88 signaling and cellular metabolism.

In addition to TCR-induced cell activation, TLR2- and TLR7-driven TCR-independent innate activation of T cells occurs independent of glycolysis. Salerno et al. reported that T cells use both aerobic glycolysis and mitochondrial respiration to produce energy during T-cell activation, and memory cells respond to TLR stimulation by fueling internally stored glucose for metabolic demands [59]. The direct response of memory cells to TLRs requires mitochondrial respiration, leading to significant but limited production of IFN-γ. This innate activation of the intrinsic TLR-signaling pathway in memory T cells may play a role in nonspecific surveillance against unrelated infections.

### Transcriptional and post-transcriptional regulation of cytokine production by TLRs

The production of antiviral cytokines, such as IFN-γ, is regulated at multiple levels, such as transcriptionally, epigenetically, and post-transcriptionally. The TCR signal-induced transcription factor T-bet is well characterized as the central regulator that promotes IFN-γ mRNA transcription. Significantly upregulated expression of T-bet is induced by TLR2 and TLR7 costimulation in synergism with TCR engagement [50, 56, 67]. The PI3K-Akt pathway is involved in TLR-induced T-bet mRNA expression and IFN-γ production [50, 66]. The stability of IFN-γ mRNA is related to adenylate uridylate-rich elements (AREs) located in the 3' untranslated region (UTR) of IFN-γ mRNA [68]. The costimulatory molecules CD28 and LFA are beneficial for stabilizing cytokine mRNAs and improving the frequencies of cytokine-producing T cells [44, 45]. Similarly, costimulation with a TLR2 agonist, but not a TLR7 agonist, enhances IFN-γ mRNA stability and prolongs the half-life of IFN-γ mRNA [59]. It is still not clear whether TLR2-induced stabilization of IFN-γ mRNA is related to AREs. Moreover, mTOR is an important modulator of the IFN-γ protein translation rate in antigen-experienced T cells. TLR2 engagement not only enhances the transcription and stability of IFN-γ mRNA but also improves the translation of IFN-γ [48, 59]. The mechanisms of TLR-MyD88 signaling induced by TLR2 and TLR7 remain to be further clarified, but current understanding involves them exhibiting distinct activity in the post-transcriptional regulation of IFN-γ mRNA. It is also undefined whether other effector cytokines of T cells, including TNF-α and IL-2, are regulated by similar mechanisms.

**Fig. 1:**
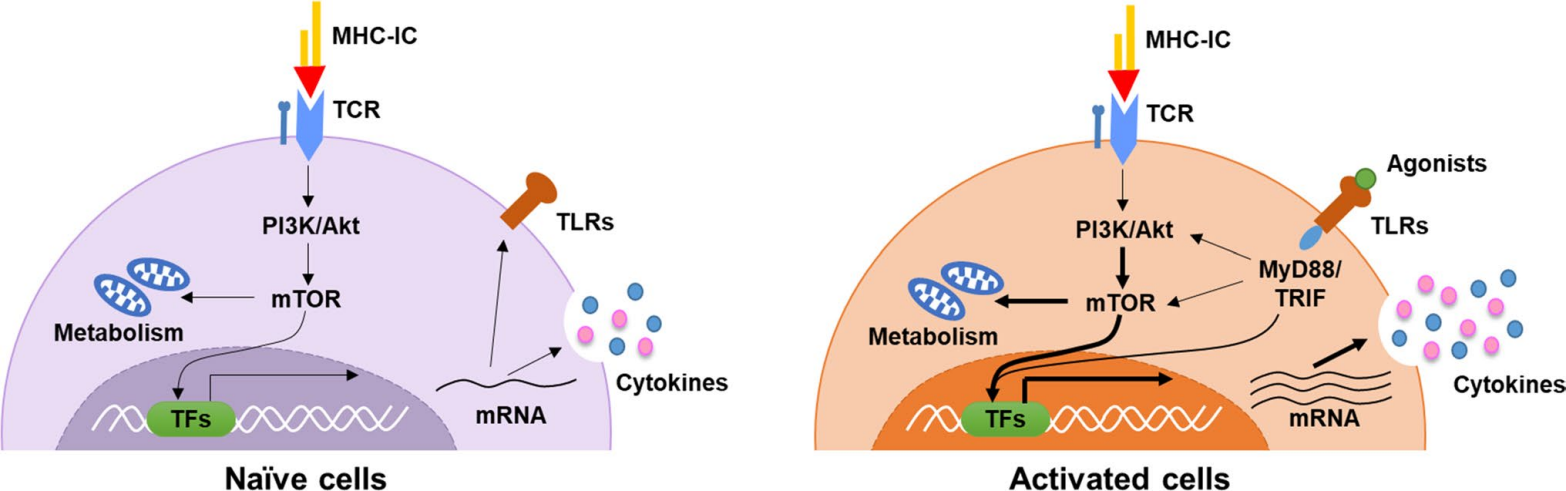
Interaction of TLR and TCR signaling pathways in T cells. Activation of naïve CD4 + and CD8 + T cells is initiated by recognition of the TCR and MHC/peptide complex. TCR signaling is transmitted by the CD3 molecule and then i.a. activates the PI3K/Akt/mTOR pathway, leading to the reprogramming of energy metabolism and activation of transcription factors, such as NF-κB, NFAT, and AP1. These transcription factors control the differentiation of T cells, the downstream production of cytokines, and upregulate the expression of TLRs (left panel). In the activated T cells, engagement of TLR agonists and TLRs initiates downstream signal cascades by recruiting adaptor proteins such as MyD88 or TRIF, leading to enhanced activation of PI3K/Akt/mTOR pathway, upregulated energy metabolism, and activates the transcription factors such as NF-κB and IRF4 to regulate the production of proinflammatory cytokines and chemokines (right panel). TLRs expressed in CD4 + and CD8 + T cells serve as costimulatory molecules in enhancing TCR signal-induced T-cell activation and function survival

Taken together, intrinsic TLR signaling, especially that activated by TLR2 and TLR7, regulates the activation and function of T cells by stimulating additional pathways for cytokine production at the levels of mRNA transcription, mRNA stability, translation and energy supply (Fig. 1). However, the signaling pathways and cascades involved in either TCR-dependent or TCR-independent TLR engagement are still not entirely understood. Moreover, the roles of intrinsic TLR signaling are primarily studied in the context of naïve animals or cells that are not undergoing infection, while expression of TLRs and intracellular signaling molecules in T cells is variable during acute and chronic viral infection. It is also important to investigate the interaction between TLR signaling and TCR signaling at the different stages of infection to further elucidate the role of TLR signaling in regulating the adaptive response during viral infection.

## Direct regulation of B-cell activation and function by the intrinsic TLR signaling pathway

It has been proposed that in vitro activation of human naïve B cells requires BCR crosslinking by antigen and CD40 stimulation from helper T cells to undergo cellular activation, proliferation, class-switch recombination (CSR), maturation of antibody affinity, and plasma cell differentiation [36]. Mature B cells initially secrete IgM or IgD antibodies after activation, and CSR enables B cells to switch to express different classes of antibodies, including IgG, IgA or IgE, that exhibit distinct effector functions [69]. TLR1/2, TLR2/6, TLR7, and TLR9, but not TLR3, TLR4 or TLR5 agonists, provide additional signals to human naïve B cells, which is beneficial for B-cell activation and antibody production [36]. Several studies have suggested that B-cell intrinsic TLR signaling synergizes with BCR signaling to induce CSR by upregulating the expression of activation-induced cytidine deaminase (AID) [70, 71]. Recently, two studies from different groups demonstrated that costimulation of B-cell intrinsic TLR7 and BCR increased somatic hypermutation, memory B-cell formation, and secondary antibody response to antigens [72, 73]. These studies imply that the B-cell intrinsic signaling pathway plays an important role both in the activation of B cells and secretion of antibodies from B cells or plasma cells.

An interesting question is whether the B-cell-intrinsic TLR/MyD88 signaling pathway is required for the induction of antibody responses to proteins or pathogens in vivo. Several groups have addressed this question and obtained controversial results. One earlier study concluded that activation of TLRs in B cells is necessary for antibody responses to T-dependent antigens [74]. However, two subsequent studies demonstrated that B-cell-intrinsic MyD88 signaling is not required to generate T-dependent antigen-specific antibody responses, but such signals can augment early antibody production, influence CSR and promote differentiation of memory B cells into plasma cells [75, 76]. Recently, using mice with either DCs or B cells with conditional MyD88 knockout, Hou et al. and colleagues demonstrated that the antibody response against purified antigen with different forms of CpG required DCs but not B-cell-intrinsic Myd88. In contrast, antigen-specific IgG responses to immunization with CpG DNA incorporated in virus-like particles (VLPs) that were derived from the Qβ bacteriophage largely depended on MyD88 expression in B cells but not DCs [77]. Consistently, the influenza virus-specific IgG response was also impaired in B-cell-specific MyD88-deficient mice following immunization with inactivated H1N1 virus [77]. Further study from the same group clarified that B-cell-intrinsic MyD88 signaling significantly enhanced the initial proliferation of Ag-specific B cells and germinal center (GC) responses and led to preferential isotype switching to IgG2a/c in a Qβ bacteriophage VLP-immunized mouse model [78]. These results seem to indicate the in vivo importance of the B-cell-intrinsic TLR signaling pathway in the generation of antiviral humoral immunity against viral infection.

In addition to their role of antibody producing cells, B cells can serve as professional APCs to induce the activation and differentiation of CD4 + T cells, as well as for memory maintenance [79]. Studies demonstrated that antigen presenting B cells were necessary and sufficient to prime cognate CD4 + T cells and induce their differentiation of follicular T helper cells independent of DCs in the LCMV and malaria infection model [80, 81]. Increasing number of studies demonstrated that B cells loaded with tumor antigens may be used as cell-based immunotherapy to stimulate antitumor CD4 + and CD8 + T-cell response [82, 83]. Stimulation of B cells with TLR ligands or by viral infection has been shown to enhance antigen presentation function by upregulating costimulatory molecules CD80, CD86, and CD40, as well as MHC molecules [84, 85].

## TLRs improve in vivo antiviral adaptive immune responses

The majority of TLRs have been reported to be involved in controlling viral infection through different mechanisms. The primary antiviral activities of TLRs, such as TLR2, TLR3, TLR7, and TLR9, are well characterized in multiple infectious diseases and are mediated by activating the innate immune response in the infected cells, thereafter producing antiviral cytokines such as IFN-I and TNF-α [86–88]. In the past two decades, studies have highlighted that the activation of TLRs is beneficial for long-term viral control by improving viral-specific T-cell or B-cell immune responses in vivo [50, 89, 90]. Ma et al. reported that deficiency of either MyD88 or TLR2/4 results in prolonged viral replication along with reduced quality and quantity of HBV-specific T cells in the liver in an HBV hydrodynamically injected mouse model [90]. Cell-specific deletion of MyD88 in B cells results in a significantly reduced antibody response and dramatic increase in the viral infectious center in a Friend virus-infected mouse model [87]. A number of publications have shown that triggering TLR2, TLR3, TLR7/8 or TLR9 suppresses viral replication in vivo by enhancing viral-specific immunity in HBV, HCV, HIV, and other viral infectious diseases [10, 86, 89–96].

**Fig. 2:**
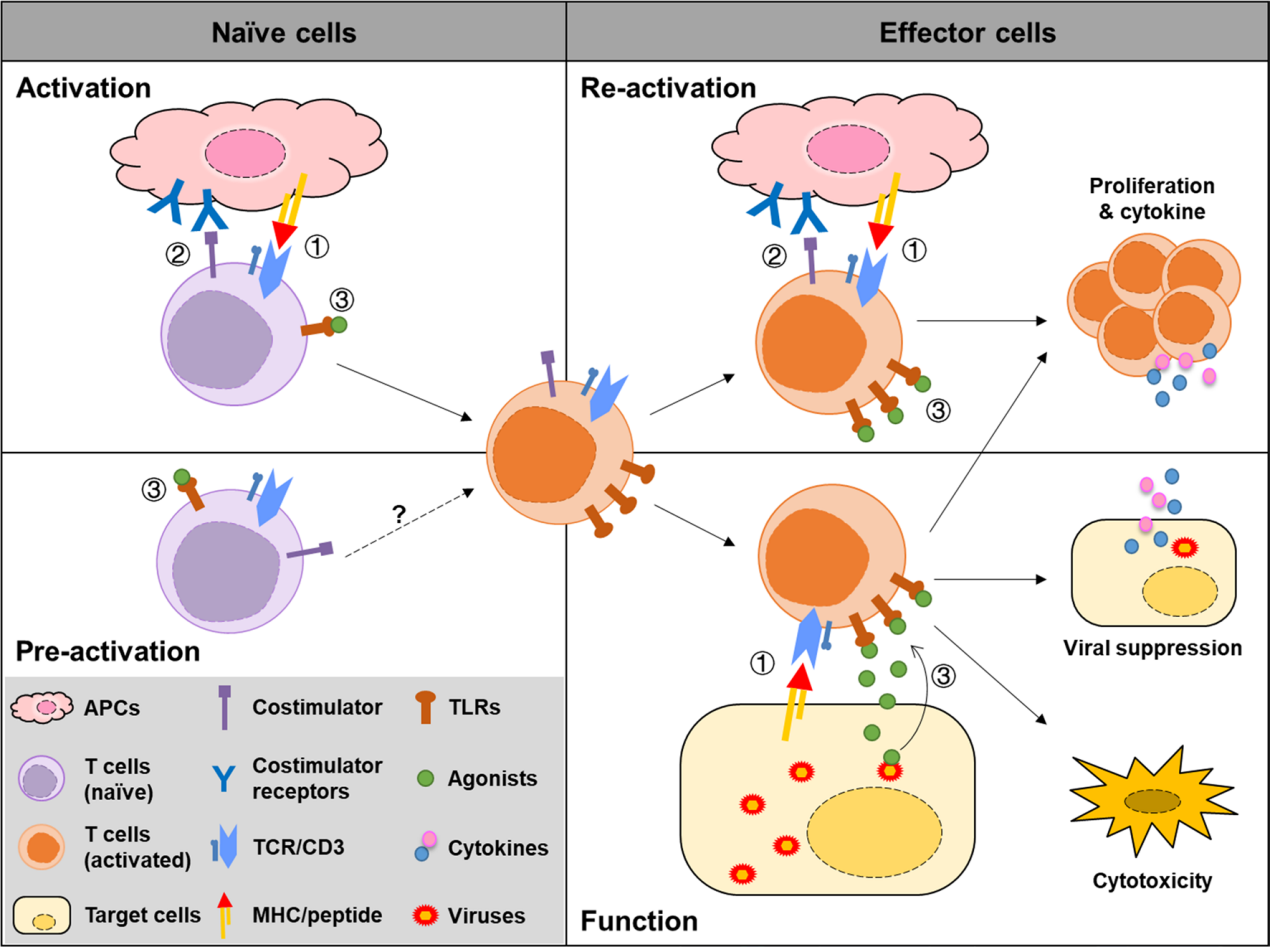
Activation of T cells by TLR engagement. Activation of naïve T cells requires at least two signals by interaction with APCs, including (1) The primary TCR signaling that was initiated by recognition of the MHC/peptide complex or antigen; (2) Secondary signals mediated by the interaction of costimulatory with their ligands. Primary and secondary signaling induces the activation of naïve cells and improve the expression of TLRs on the T cells. In addition, (3) engagement of TLRs with their agonists provides additional signals to enhance TCR signaling (upper left panel). TLR signaling alone may induce partial activation of naïve T cells, but more conclusive evidence is needed (lower left panel). Activated or effector T cells undergo reactivation upon recognizing viral-specific antigens presented on APCs and activates the TCR signaling, costimulatory signaling and TLR signaling from TLR agonists derived from pathogens, resulting in rapid and vigorous proliferation and cytokine production (upper right panel). At the site of infection, effector T cells recognize the antigens on the target cells which lack of the costimulatory molecules. Virus-derived TLR agonists engage with TLRs in T cells and provide alternative secondary signals for the T cell proliferation, cytokine production and cytotoxic activities of T cells (lower right panel)

Regulation of the in vivo antiviral adaptive immune response is much more complicated due to the interaction of different cell types in response to TLR engagement. One of the important functions of TLRs is to recruit immune cells, including T cells, B cells, monocytes, NK cells and neutrophils, to the infected site by stimulating the infected cells and likely neighboring cells to produce proinflammatory cytokines and chemokines [86, 97]. Wu et al. reported that poly(I:C)-induced HBV clearance was significantly impaired in CXCR3-deficient mice, indicating that the stimulation and recruitment of T cells into the liver are critical for HBV clearance in the HBV replicative mouse model [86]. Meanwhile, stimulation of TLRs in immune cells plays an equally important role in inducing and maintaining sustained antiviral adaptive immune responses against both ongoing infection and possible reinfection. TLR-stimulated APCs, including DCs and macrophages in the peripheral lymphoid organs, regulate the activation and differentiation of T cells at the priming and maturation stages of immune responses, while TLRs stimulate immune regulatory cells in infected tissues to modulate the function and fate of infiltrated viral-specific T cells [98]. For example, intrahepatic LSECs exert a positive role in improving the amount, function and proliferation of intrahepatic CD8 + T cells in response to TLR2 or TLR5 agonist stimulation [52, 99], while TLR2-stimulated KCs exhibit enhanced suppressive activity against CD8 + T cells by secreting IL-10 [100]. Moreover, TLRs from pathogens may directly provide costimulatory signals in the absence of traditional costimulatory molecules. Hepatocytes, which have strong immune inhibitory activities due to a lack of costimulatory factors, such as CD40, CD28 or ICOS, display reversed immune regulatory activities after TLR stimulation or viral infection, leading to significantly improved T-cell activation [52]. This may be at least partially related to TLRs remaining in the culture system or hepatocytes. Therefore, PAMPs derived from pathogens may benefit the activation and maintenance of T-cell responses during the interaction of viral-specific T cells, targeting cells independent of the expression of costimulatory molecules (Fig. 2).

Interactions between the TLR signaling pathway and other costimulatory factors play a role in regulating infection-induced inflammation. The TNF superfamily member 4-1BB ligand (4-1BBL) was reported to play not only an essential role in sustaining the expression of proinflammatory cytokines during macrophage activation in LPS-induced sepsis [101, 102] but also a central role in regulating the costimulatory effects of TLR1/2 signaling in T cells in a melanoma tumor mouse model [103]. Zahm et al. reported that innate immune activation of TLR1/2, TLR7, and TLR9 in T cells led to decreased expression of PD-1 on antigen-activated CD8 + T cells and thus improved antitumor immunity [29]. Chodisetti et al. also found that TLR-2 signaling is beneficial for the ability of chronically stimulated Th1 cells by improving the expression of T-bet, IL-2, BCL-1 and suppressing the expression of PD-1 and LAG-3, thus reducing lung pathology in a chronic infection model of tuberculosis [56]. These results indicate that the interaction of TLRs with immune checkpoints plays roles in regulating antitumor and antibacterial immune responses, which requires further investigation in viral infection models.

Recently, the roles of TLR7 and TLR9 in the effector function of B cells in patients with systemic lupus erythematosus have been reviewed [104]. However, the contribution of the B-cell-intrinsic TLR signaling pathway to antiviral humoral immunity during viral infection has not been studied or discussed extensively. Using an influenza-infected mouse model, Heer et al. demonstrated that MyD88 and TLR7 are not critical for the initiation of adaptive T-cell responses against influenza infection, but they do regulate anti-influenza B-cell antibody isotype switching through both direct and indirect effects on B cells. Specifically, CD40-CD40L interactions and TLR signaling on B cells result in proliferation and initiate IgG1 and IgG2a/c class switching, whereas TLR-induced type I IFN production fine-tunes the antiviral response, decreasing IgG1 and increasing IgG2a/c [105]. Several studies further demonstrated the important role of TLR7 in the development of the antiviral humoral immune response through modulation of the GC B-cell response in a mouse model with acute viral infection, such as influenza virus A [106], rabies virus [107] and enterovirus 71 [108].

Early studies suggested that B cells play an essential role in the clearance of persistent viral infection through antibody production and induction of a competent CD4 T-cell help response in a chronic lymphocytic choriomeningitis virus (LCMV)-infected mouse model [109–111]. Deficiency in TLR7 led to a significant decrease in LCMV-specific antibodies in this model, which correlated with diminished GC B-cell formation and a reduction in plasma cells. The LCMV-specific CD4 + and CD8 + T cell responses were also functionally impaired and produced less cytokines and granzyme B due to both intrinsic and environmental deficiency of TLR7, though there were higher frequencies of virus-specific T cells in the spleen [112]. Further study demonstrated that B-cell-intrinsic TLR7 is sufficient to significantly impact antibody responses in mice during chronic LCMV infection. This effect was independent of T follicular helper cells but was attributed to the qualitative effect of TLR signaling on the GC B-cell response, which later promoted the generation of plasma cells [113]. In another chronic retroviral infection mouse model, deletion of MyD88 in DCs had little effect on the immune control of Friend virus (FV), while B-cell-specific deletion of MyD88 caused a dramatic increase in viral infectious centers and a significantly reduced antibody response, indicating that B-cell-intrinsic TLR signaling plays a crucial role in viral control [87]. B-cell-intrinsic TLR7 was found to be required for the development of an effective antibody response against the virus by enhancing the GC B-cell response [87]. Interestingly, activation of TLR7 in memory CD4 + and CD8 + T cells led to secretion of IFN-γ, which synergistically with TLRs induce T-bet expression and IgG2a/c isotype switching in B cells [58]. This study revealed a surprised mechanism of crosstalk between T and B cells through the intrinsic TLR signal pathway. These studies suggest that the B-cell-intrinsic TLR signaling pathway is essential for B cells to control and terminate acute or chronic viral infection in murine models. However, it is difficult to directly verify this point in human natural viral infection. It is well known that B cells play central roles in the immune control of many acute or chronic viral infections in humans, such as influenza virus, severe acute respiratory syndrome coronavirus 2 (SARS-CoV-2), HBV and human immunodeficiency virus (HIV) [114–117]. Therefore, it is rational and encouraged to design immune therapy strategies targeting the B-cell-intrinsic TLR signaling pathway to prevent and treat viral infectious diseases in humans.

Conclusively, in addition to the direct stimulation of T-cell and B-cell intrinsic TLR signaling pathways, regulation of the TLR signaling pathway on viral-specific T/B cells in vivo exerts a comprehensive effect involving multiple factors. It is difficult to distinguish the individual contributions of those TLR-induced in vivo mechanisms in controlling the virus. Cell-specific gene modification may provide more definitive evidence, which requires a precise experimental design and further investigation.

## Presumed clinical use of TLR against viral infection

Based on the impressive results of antiviral therapies of TLRs in in vitro studies and in vivo animal models, there are a number of promising compounds targeting TLRs to treat viral infectious diseases [91, 92, 118]. Few of these compounds are antagonists that reduce the TLR signaling-related overactivation of the immune response and thus suppress immunopathology. For example, the TLR4 antagonist EB05 was designed to block the interaction between TLR4 on the innate immune cells and the DAMPs produced by viral-mediated cell damage, such as S100A8/A9, HMGB1, and oxidized phospholipids, and to ameliorate the cytokine storm and acute respiratory distress syndrome induced by COVID-19 (Table 2). Classically, the majority of these compounds are agonists that activate the TLR signaling pathway (Table 2). Strategies targeting T- and B-cell-intrinsic TLR signaling pathways are rational and promising for designing prophylactic and therapeutic vaccines against viral infectious diseases [10, 119–121]. TLR-based adjuvants have been proven to be efficient T- and B-cell activators and have been used in HBV, human papilloma virus (HPV), and herpes zoster virus (HZV) prophylactic vaccines [122–124]. VPLs derived from the Qβ bacteriophage (Qβ-VPL) selectively activated the B-cell-intrinsic TLR signaling pathway and promoted antibody production in an immunized mouse model [77, 78]. Further study found that Qβ-VPL could be used as a carrier for vaccines that utilized antigen-specific B cells as dominant antigen presenting cells to activate and promote the development of the T follicular helper cell response [125]. Importantly, using this vaccination strategy, the same group designed and constructed a COVID-19 vaccine candidate that induced robust neutralizing antibodies in both mice and nonhuman primates (NHPs). Furthermore, viral clearance was accelerated in the vaccinated group in a virus challenge experiment in the NHP model [126]. The VLP-based platform has been utilized by several studies in the development of preventive vaccines against COVID-19 and other viral diseases, and the efficacy and advantages of these vaccines in preclinical experiments and clinical trials have been reviewed extensively elsewhere [127–129].

**Table 2:** TLR-targeting ligands in clinical trials or clinical use for viral infection

Target	Ligand	Virus	Type	Phase	NCT number
TLR1/2	XS15	COVID-19	Adjuvant	Phase 1	NCT04546841
TLR4	MPL	HZV	Adjuvant	Approved	GSK (Shingrix)
		HBV	Adjuvant	Approved	GSK (Fendrix)
TLR5	VAX125	Influenza	Adjuvant	Phase 2	NCT00966238
	VAX102	Influenza	Adjuvant	Phase 1	NCT00603811
TLR7/8	Imiquimod	HBV/IBD	Adjuvant	Phase 2/3	NCT04083157
	Imiquimod	Influenza	Adjuvant	Phase 3	NCT02103023
	Resiquimod	Influenza	Adjuvant	Phase 1	NCT01737580
	3M-052-AF	HIV	Adjuvant	Phase 1	NCT04177355
TLR9	CpG 1018	HBV	Adjuvant	Approved	GSK (Cervarix)
	CpG 1018	HZV	Adjuvant	Phase 1	NCT05245838
	CPG 7909	HIV	Adjuvant	Phase 1/2	NCT00562939
TLR3	Poly-ICLC	HIV	Drug	Phase 1	NCT02071095
TLR4	TriMix	HIV	Drug	Phase 2	NCT02888756
TLR7/8	Imiquimod	HPV	Drug	Approved	3M Pharma
	RO7020531	CHB	Drug	Phase 2	NCT04225715
	TQ-A3334	CHB	Drug	Phase 2	NCT04180150
	GS-9620	CHB	Drug	Phase 2	NCT02166047
	GS-9620	HIV	Drug	Phase 2	NCT04364035
	GS-9688	CHB	Drug	Phase 2	NCT03491553 NCT03615066
	HRS9950	CHB	Drug	Phase 1	NCT04464733
	SLGN	CHB	Drug	Phase 2	NCT05045261
TLR9	SD-101	CHB	Drug	Phase 1	NCT00823862
	IMO-2125	HCV	Drug	Phase 1	NCT00728936
	Lefitolimod	HIV	Drug	Phase 1/2	NCT04357821
IRAK4 (inhibitor)	PF-06650833	COVID-19	Drug	Phase 2	NCT04933799
TLR3 (antagonist)	TAO1	Common cold Influenza	Drug	Phase 1/2	NCT01651715
TLR4 (antagonist)	EB05	COVID-19	Drug	Phase 2	NCT04401475
	ApTOLL	COVID-19	Drug	Phase 1	NCT05293236

All data derived from the database of ClinicalTrials.gov (https://clinicaltrials.gov/)

Abbreviations: *HZV* herpes zoster virus, *HBV* hepatitis B virus, *IBD* inflammatory bowel disease, *HIV* human immunodeficiency virus, *HPV* human papilloma virus, *CHB* chronic hepatitis B infection, *HCV* hepatitis C virus, *TLR* toll-like receptor

A challenge is to develop potential TLR-based adjuvants for therapeutic vaccines to ameliorate the immunologic microenvironment and to stimulate proinflammatory cytokines for the restoration of T- and B-cell immune responses. Treatment with TLR agonists alone aims to induce antiviral factors such as ISGs to suppress the virus, and TLR signaling-induced proinflammatory cytokines are intended to benefit the activation of antiviral immune cells. These agonists, including the TLR9 agonist SD-101 and the TLR7 agonists imiquimod, GS9620 and RO7020531, are now in clinical trials. However, GS9620, one of the most promising candidates, resulted in limited improvement in viral DNA control, serum HBsAg reduction and HBeAg seroconversion when administered alone in CHB patients during a phase 2 clinical trial despite positive results in chronically infected chimpanzees [93, 95]. In recent years, researchers have tended to use TLR agonists in combination with other antiviral drugs to treat chronic viral infectious diseases. For example, GS9620 and RO7020531 are in new clinical trials in combination with nucleos(t)ide analogues to treat CHB, and lefitolimod is used in combination with ATI to treat HIV.

The other feasible hypothesis is to combine TLR agonists with checkpoint inhibitors to restore exhausted viral-specific T-cell responses. The combination of the TLR9 agonist ODN1826 with either CTLA-4 or PD-1 blockade showed improved intertumoral CD8 + T-cell responses and suppressed tumor growth in a melanoma mouse model [130]. Similarly, TLR7/8 agonist-based tumor vaccines also demonstrated better therapeutic efficacy in combination with PD-L1 blockade in murine tumor models [131, 132]. This may be one of the strategies to alleviate the immunosuppression and promote the functional recovery of viral-specific T cells during chronic viral infection. Gene-modified T cells expressing specific TCRs afford abundant and functional antigen-specific T cells by in vitro technologies [133, 134]. Tumor-specific CAR T cells have generated four generations. In the new design of CAR T cells, the TIR domain of TLR2 or the TLR adaptor molecule MyD88 is employed in the CARs [18]. The TLR pathway signaling domains in cooperation with other fuses show synthetic effects on improving the effector function and reducing the exhaustion of T cells [135, 136]. TCR-transgenic T cells have shown efficient viral control activities in virus-infected mice, such as in chronic HBV replicating mouse models, HBV transgenic mice, and chronic LCMV infected mouse models [137, 138]. Development and application of TCR-transgenic T cells in infectious diseases is much slower than that in tumors. The experience from CAR T cells suggests that TCR-transgenic T cells containing the TLR signaling pathway domains may represent an improved regimen for T-cell therapy of chronic infections. However, the consequent issues of immune overactivation and immunopathology require more attention and personalized treatment.

Taken together, emerging animal experiments, preclinical studies, and clinical trials represent a promising potential of TLR-targeting compounds in inducing prophylactic and therapeutic immune responses against viral infectious diseases. The combination treatment of TLR agonists together with other antiviral and immunomodulatory drugs represents an important topic for future clinical experiments. The aim is to stimulate an effective T- and B-cell immune response during the viral suppression period and, therefore, obtain long-term immune protection after withdrawal of antiviral drugs. With the development of new techniques, including computer-aided design, next-generation sequencing, omics data, nanotechnology and big data analysis, we hope to generate more effective TLR-vaccine combination regimens and TLR-antiviral drug combination strategies to improve the protective viral-specific acquired immune responses.

## Data Availability

All cited articles in the current study are available in the public database.

## References

[1] Kemming J, Thimme R, Neumann-Haefelin C (2020). Adaptive immune response against hepatitis C virus. Int J Mol Sci.

[2] Rossignol E, Alter G, Julg B (2021). Antibodies for human immunodeficiency virus-1 cure strategies. J Infect Dis.

[3] Thimme R, Wieland S, Steiger C (2003). CD8(+) T cells mediate viral clearance and disease pathogenesis during acute hepatitis B virus infection. J Virol.

[4] Collins DR, Gaiha GD, Walker BD (2020). CD8(+) T cells in HIV control, cure and prevention. Nat Rev Immunol.

[5] Bertoletti A, Kennedy PT (2015). The immune tolerant phase of chronic HBV infection: new perspectives on an old concept. Cell Mol Immunol.

[6] Gehring AJ, Protzer U (2019). Targeting innate and adaptive immune responses to cure chronic HBV infection. Gastroenterology.

[7] Rha MS, Shin EC (2021). Activation or exhaustion of CD8(+) T cells in patients with COVID-19. Cell Mol Immunol.

[8] Takeda K, Kaisho T, Akira S (2003). Toll-like receptors. Annu Rev Immunol.

[9] Li C, Kuang WD, Qu D, Wang JH (2016). Toll-interacting protein inhibits HIV-1 infection and regulates viral latency. Biochem Biophys Res Commun.

[10] Ma Z, Zhang E, Yang D, Lu M (2015). Contribution of Toll-like receptors to the control of hepatitis B virus infection by initiating antiviral innate responses and promoting specific adaptive immune responses. Cell Mol Immunol.

[11] Iwasaki A, Medzhitov R (2004). Toll-like receptor control of the adaptive immune responses. Nat Immunol.

[12] Rojas-Sanchez L, Zhang E, Sokolova V (2020). Genetic immunization against hepatitis B virus with calcium phosphate nanoparticles in vitro and in vivo. Acta Biomater.

[13] Cebula M, Riehn M, Hillebrand U (2017). TLR9-mediated conditioning of liver environment is essential for successful intrahepatic immunotherapy and effective memory recall. Mol Ther.

[14] Rammensee HG, Wiesmuller KH, Chandran PA (2019). A new synthetic toll-like receptor 1/2 ligand is an efficient adjuvant for peptide vaccination in a human volunteer. J Immunother Cancer.

[15] Matsumoto M, Takeda Y, Seya T (2020). Targeting Toll-like receptor 3 in dendritic cells for cancer immunotherapy. Expert Opin Biol Ther.

[16] Chen Z, Cheng Y, Xu Y (2008). Expression profiles and function of Toll-like receptors 2 and 4 in peripheral blood mononuclear cells of chronic hepatitis B patients. Clin Immunol.

[17] Moradi M, Tabibzadeh A, Javanmard D (2020). Assessment of key elements in the innate immunity system among patients with HIV, HCV, and coinfections of HIV/HCV. Curr HIV Res.

[18] Nouri Y, Weinkove R, Perret R (2021). T-cell intrinsic Toll-like receptor signaling: implications for cancer immunotherapy and CAR T-cells. J Immunother Cancer.

[19] Reynolds JM, Dong C (2013). Toll-like receptor regulation of effector T lymphocyte function. Trends Immunol.

[20] Kawai T, Akira S (2010). The role of pattern-recognition receptors in innate immunity: update on Toll-like receptors. Nat Immunol.

[21] Akira S, Takeda K (2004). Toll-like receptor signalling. Nat Rev Immunol.

[22] Hornung V, Rothenfusser S, Britsch S (2002). Quantitative expression of toll-like receptor 1–10 mRNA in cellular subsets of human peripheral blood mononuclear cells and sensitivity to CpG oligodeoxynucleotides. J Immunol.

[23] Caron G, Duluc D, Fremaux I (2005). Direct stimulation of human T cells via TLR5 and TLR7/8: flagellin and R-848 up-regulate proliferation and IFN-gamma production by memory CD4+ T cells. J Immunol.

[24] McCarron M, Reen DJ (2009). Activated human neonatal CD8+ T cells are subject to immunomodulation by direct TLR2 or TLR5 stimulation. J Immunol.

[25] Mansson A, Adner M, Cardell LO (2006). Toll-like receptors in cellular subsets of human tonsil T cells: altered expression during recurrent tonsillitis. Respir Res.

[26] Gelman AE, Zhang J, Choi Y, Turka LA (2004). Toll-like receptor ligands directly promote activated CD4+ T cell survival. J Immunol.

[27] Caramalho I, Lopes-Carvalho T, Ostler D (2003). Regulatory T cells selectively express toll-like receptors and are activated by lipopolysaccharide. J Exp Med.

[28] Cottalorda A, Verschelde C, Marcais A (2006). TLR2 engagement on CD8 T cells lowers the threshold for optimal antigen-induced T cell activation. Eur J Immunol.

[29] Zahm CD, Colluru VT, McIlwain SJ, Ong IM, McNeel DG (2018). TLR stimulation during T-cell activation lowers PD-1 expression on CD8(+) T cells. Cancer Immunol Res.

[30] Komai-Koma M, Jones L, Ogg GS, Xu D, Liew FY (2004). TLR2 is expressed on activated T cells as a costimulatory receptor. Proc Natl Acad Sci USA.

[31] Huang YW, Lin SC, Wei SC (2013). Reduced Toll-like receptor 3 expression in chronic hepatitis B patients and its restoration by interferon therapy. Antivir Ther.

[32] Xu C, Lu Y, Zheng X (2017). TLR2 expression in peripheral CD4+ T cells promotes Th17 response and is associated with disease aggravation of hepatitis b virus-related acute-on-chronic liver failure. Front Immunol.

[33] Bekeredjian-Ding I, Jego G (2009). Toll-like receptors–sentries in the B-cell response. Immunology.

[34] Browne EP (2012). Regulation of B-cell responses by Toll-like receptors. Immunology.

[35] Mansson A, Adner M, Hockerfelt U, Cardell LO (2006). A distinct Toll-like receptor repertoire in human tonsillar B cells, directly activated by PamCSK, R-837 and CpG-2006 stimulation. Immunology.

[36] Ruprecht CR, Lanzavecchia A (2006). Toll-like receptor stimulation as a third signal required for activation of human naive B cells. Eur J Immunol.

[37] Bourke E, Bosisio D, Golay J, Polentarutti N, Mantovani A (2003). The toll-like receptor repertoire of human B lymphocytes: inducible and selective expression of TLR9 and TLR10 in normal and transformed cells. Blood.

[38] Genestier L, Taillardet M, Mondiere P (2007). TLR agonists selectively promote terminal plasma cell differentiation of B cell subsets specialized in thymus-independent responses. J Immunol.

[39] Gururajan M, Jacob J, Pulendran B (2007). Toll-like receptor expression and responsiveness of distinct murine splenic and mucosal B-cell subsets. PLoS One.

[40] Marshall-Clarke S, Downes JE, Haga IR (2007). Polyinosinic acid is a ligand for toll-like receptor 3. J Biol Chem.

[41] Sato S, Sanjo H, Takeda K (2005). Essential function for the kinase TAK1 in innate and adaptive immune responses. Nat Immunol.

[42] Chang WL, Coro ES, Rau FC (2007). Influenza virus infection causes global respiratory tract B cell response modulation via innate immune signals. J Immunol.

[43] Bekeredjian-Ding IB, Wagner M, Hornung V (2005). Plasmacytoid dendritic cells control TLR7 sensitivity of naive B cells via type I IFN. J Immunol.

[44] Schmitz ML, Krappmann D (2006). Controlling NF-κB activation in T cells by costimulatory receptors. Cell Death Differ.

[45] Chen L, Flies DB (2013). Molecular mechanisms of T cell co-stimulation and co-inhibition. Nat Rev Immunol.

[46] Ruterbusch M, Pruner KB, Shehata L, Pepper M (2020). In vivo CD4(+) T cell differentiation and function: revisiting the Th1/Th2 paradigm. Annu Rev Immunol.

[47] Imanishi T, Hara H, Suzuki S (2007). Cutting edge: TLR2 directly triggers Th1 effector functions. J Immunol.

[48] Salerno F, Freen-van Heeren JJ, Guislain A, Nicolet BP, Wolkers MC (2019). Costimulation through TLR2 drives polyfunctional CD8(+) T cell responses. J Immunol.

[49] Imanishi T, Unno M, Kobayashi W (2020). mTORC1 signaling controls TLR2-mediated T-cell activation by inducing TIRAP expression. Cell Rep.

[50] Geng D, Zheng L, Srivastava R (2010). When Toll-like receptor and T-cell receptor signals collide: a mechanism for enhanced CD8 T-cell effector function. Blood.

[51] Mercier BC, Cottalorda A, Coupet CA, Marvel J, Bonnefoy-Berard N (2009). TLR2 engagement on CD8 T cells enables generation of functional memory cells in response to a suboptimal TCR signal. J Immunol.

[52] Yan H, Zhong M, Yang J (2020). TLR5 activation in hepatocytes alleviates the functional suppression of intrahepatic CD8(+) T cells. Immunology.

[53] Veldhoen M, Hocking RJ, Atkins CJ, Locksley RM, Stockinger B (2006). TGFbeta in the context of an inflammatory cytokine milieu supports de novo differentiation of IL-17-producing T cells. Immunity.

[54] Nyirenda MH, Sanvito L, Darlington PJ (2011). TLR2 stimulation drives human naive and effector regulatory T cells into a Th17-like phenotype with reduced suppressive function. J Immunol.

[55] Karim AF, Reba SM, Li Q, Boom WH, Rojas RE (2017). Toll like receptor 2 engagement on CD4(+) T cells promotes TH9 differentiation and function. Eur J Immunol.

[56] Chodisetti SB, Gowthaman U, Rai PK (2015). Triggering through Toll-like receptor 2 limits chronically stimulated T-helper type 1 cells from undergoing exhaustion. J Infect Dis.

[57] Imanishi T, Ishihara C, Badr Mel S (2014). Nucleic acid sensing by T cells initiates Th2 cell differentiation. Nat Commun.

[58] Rubtsova K, Rubtsov AV, Halemano K (2016). T cell production of IFNγ in response to TLR7/IL-12 stimulates optimal B cell responses to viruses. PLoS One.

[59] Salerno F, Guislain A, Cansever D, Wolkers MC (2016). TLR-mediated innate production of IFN-γ by CD8+ T cells is independent of glycolysis. J Immunol.

[60] Cottalorda A, Mercier BC, Mbitikon-Kobo FM (2009). TLR2 engagement on memory CD8(+) T cells improves their cytokine-mediated proliferation and IFN-γ secretion in the absence of Ag. Eur J Immunol.

[61] MacIver NJ, Michalek RD, Rathmell JC (2013). Metabolic regulation of T lymphocytes. Annu Rev Immunol.

[62] Wang R, Green DR (2012). Metabolic checkpoints in activated T cells. Nat Immunol.

[63] Schurich A, Pallett LJ, Jajbhay D (2016). Distinct metabolic requirements of exhausted and functional virus-specific CD8 T cells in the same host. Cell Rep.

[64] Boussiotis VA (2016). Molecular and biochemical aspects of the PD-1 checkpoint pathway. N Engl J Med.

[65] Fisicaro P, Barili V, Montanini B (2017). Targeting mitochondrial dysfunction can restore antiviral activity of exhausted HBV-specific CD8 T cells in chronic hepatitis B. Nat Med.

[66] Li Q, Yan Y, Liu J (2019). Toll-like receptor 7 activation enhances CD8+ T cell effector functions by promoting cellular glycolysis. Front Immunol.

[67] Zhang E, Ma Z, Li Q (2019). TLR2 stimulation increases cellular metabolism in CD8(+) T cells and thereby enhances CD8(+) T cell activation, function, and antiviral activity. J Immunol.

[68] Freen-van Heeren JJ, Popovic B, Guislain A, Wolkers MC (2020). Human T cells employ conserved AU-rich elements to fine-tune IFN-γ production. Eur J Immunol.

[69] Chen Z, Wang JH (2021). How the signaling crosstalk of B cell receptor (BCR) and co-receptors regulates antibody class switch recombination: a new perspective of checkpoints of BCR signaling. Front Immunol.

[70] Pone EJ, Zhang J, Mai T (2012). BCR-signalling synergizes with TLR-signalling for induction of AID and immunoglobulin class-switching through the non-canonical NF-κB pathway. Nat Commun.

[71] Pone EJ, Lou Z, Lam T (2015). B cell TLR1/2, TLR4, TLR7 and TLR9 interact in induction of class switch DNA recombination: modulation by BCR and CD40, and relevance to T-independent antibody responses. Autoimmunity.

[72] Castiblanco DP, Maul RW, Russell Knode LM, Gearhart PJ (2017). Co-stimulation of BCR and toll-like receptor 7 increases somatic hypermutation, memory B cell formation, and secondary antibody response to protein antigen. Front Immunol.

[73] Krueger CC, Thoms F, Keller E (2019). RNA and toll-like receptor 7 license the generation of superior secondary plasma cells at multiple levels in a B cell intrinsic fashion. Front Immunol.

[74] Pasare C, Medzhitov R (2005). Control of B-cell responses by toll-like receptors. Nature.

[75] Gavin AL, Hoebe K, Duong B (2006). Adjuvant-enhanced antibody responses in the absence of toll-like receptor signaling. Science.

[76] Meyer-Bahlburg A, Khim S, Rawlings DJ (2007). B cell intrinsic TLR signals amplify but are not required for humoral immunity. J Exp Med.

[77] Hou B, Saudan P, Ott G (2011). Selective utilization of Toll-like receptor and MyD88 signaling in B cells for enhancement of the antiviral germinal center response. Immunity.

[78] Tian M, Hua Z, Hong S (2018). B cell-intrinsic MyD88 signaling promotes initial cell proliferation and differentiation to enhance the germinal center response to a virus-like particle. J Immunol.

[79] Ghosh D, Jiang W, Mukhopadhyay D, Mellins ED (2021). New insights into B cells as antigen presenting cells. Curr Opin Immunol.

[80] Arroyo EN, Pepper M (2020). B cells are sufficient to prime the dominant CD4+ Tfh response to plasmodium infection. J Exp Med.

[81] Barnett LG, Simkins HM, Barnett BE (2014). B cell antigen presentation in the initiation of follicular helper T cell and germinal center differentiation. J Immunol.

[82] Page A, Hubert J, Fusil F, Cosset FL (2021). Exploiting B cell transfer for cancer therapy: engineered B cells to eradicate tumors. Int J Mol Sci.

[83] Wennhold K, Shimabukuro-Vornhagen A, Theurich S, von Bergwelt-Baildon M (2013). CD40-activated B cells as antigen-presenting cells: the final sprint toward clinical application. Expert Rev Vaccines.

[84] Moore TC, Messer RJ, Gonzaga LM (2019). Effects of friend virus infection and regulatory T cells on the antigen presentation function of B cells. MBio.

[85] Jiang W, Lederman MM, Harding CV (2007). TLR9 stimulation drives naive B cells to proliferate and to attain enhanced antigen presenting function. Eur J Immunol.

[86] Wu J, Huang S, Zhao X (2014). Poly(I:C) treatment leads to interferon-dependent clearance of hepatitis B virus in a hydrodynamic injection mouse model. J Virol.

[87] Browne EP (2011). Toll-like receptor 7 controls the anti-retroviral germinal center response. PLoS Pathog.

[88] Zhang X, Ma Z, Liu H (2012). Role of toll-like receptor 2 in the immune response against hepadnaviral infection. J Hepatol.

[89] Tsai A, Irrinki A, Kaur J (2017). Toll-like receptor 7 agonist GS-9620 induces HIV expression and HIV-specific immunity in cells from HIV-infected individuals on suppressive antiretroviral therapy. J Virol.

[90] Ma Z, Liu J, Wu W (2017). The IL-1R/TLR signaling pathway is essential for efficient CD8(+) T-cell responses against hepatitis B virus in the hydrodynamic injection mouse model. Cell Mol Immunol.

[91] Martinsen JT, Gunst JD, Hojen JF, Tolstrup M, Sogaard OS (2020). The use of toll-like receptor agonists in HIV-1 cure strategies. Front Immunol.

[92] Shah M, Anwar MA, Kim JH, Choi S (2016). Advances in antiviral therapies targeting toll-like receptors. Expert Opin Investig Drugs.

[93] Lanford RE, Guerra B, Chavez D (2013). GS-9620, an oral agonist of Toll-like receptor-7, induces prolonged suppression of hepatitis B virus in chronically infected chimpanzees. Gastroenterology.

[94] Dou Y, Jansen D, van den Bosch A (2020). Design of TLR2-ligand-synthetic long peptide conjugates for therapeutic vaccination of chronic HBV patients. Antiviral Res.

[95] Boni C, Vecchi A, Rossi M (2018). TLR7 agonist increases responses of hepatitis B virus-specific T cells and natural killer cells in patients with chronic hepatitis B treated with nucleos(T)Ide analogues. Gastroenterology.

[96] Lin Y, Huang X, Wu J (2018). Pre-activation of toll-like receptor 2 enhances CD8(+) T-Cell responses and accelerates hepatitis B virus clearance in the mouse models. Front Immunol.

[97] Huang LR, Wohlleber D, Reisinger F (2013). Intrahepatic myeloid-cell aggregates enable local proliferation of CD8(+) T cells and successful immunotherapy against chronic viral liver infection. Nat Immunol.

[98] Wu J, Meng Z, Jiang M (2010). Toll-like receptor-induced innate immune responses in non-parenchymal liver cells are cell type-specific. Immunology.

[99] Liu J, Jiang M, Ma Z (2013). TLR1/2 ligand-stimulated mouse liver endothelial cells secrete IL-12 and trigger CD8+ T cell immunity in vitro. J Immunol.

[100] Liu J, Yu Q, Wu W (2018). TLR2 stimulation strengthens intrahepatic myeloid-derived cell-mediated T cell tolerance through inducing Kupffer cell expansion and IL-10 production. J Immunol.

[101] Ma J, Bang BR, Lu J (2013). The TNF family member 4–1BBL sustains inflammation by interacting with TLR signaling components during late-phase activation. Sci Signal.

[102] Bang BR, Kim SJ, Yagita H, Croft M, Kang YJ (2015). Inhibition of 4–1BBL-regulated TLR response in macrophages ameliorates endotoxin-induced sepsis in mice. Eur J Immunol.

[103] Joseph AM, Srivastava R, Zabaleta J, Davila E (2016). Cross-talk between 4–1BB and TLR1-TLR2 signaling in CD8+ T cells regulates TLR2's costimulatory effects. Cancer Immunol Res.

[104] Fillatreau S, Manfroi B, Dorner T (2021). Toll-like receptor signalling in B cells during systemic lupus erythematosus. Nat Rev Rheumatol.

[105] Heer AK, Shamshiev A, Donda A (2007). TLR signaling fine-tunes anti-influenza B cell responses without regulating effector T cell responses. J Immunol.

[106] Jeisy-Scott V, Kim JH, Davis WG (2012). TLR7 recognition is dispensable for influenza virus A infection but important for the induction of hemagglutinin-specific antibodies in response to the 2009 pandemic split vaccine in mice. J Virol.

[107] Luo Z, Li Y, Zhou M (2019). Toll-like receptor 7 enhances rabies virus-induced humoral immunity by facilitating the formation of germinal centers. Front Immunol.

[108] Lin YL, Lu MY, Chuang CF (2020). TLR7 is critical for anti-viral humoral immunity to EV71 infection in the spinal cord. Front Immunol.

[109] Homann D, Tishon A, Berger DP (1998). Evidence for an underlying CD4 helper and CD8 T-cell defect in B-cell-deficient mice: failure to clear persistent virus infection after adoptive immunotherapy with virus-specific memory cells from muMT/muMT mice. J Virol.

[110] Bergthaler A, Flatz L, Verschoor A (2009). Impaired antibody response causes persistence of prototypic T cell-contained virus. PLoS Biol.

[111] Whitmire JK, Asano MS, Kaech SM (2009). Requirement of B cells for generating CD4+ T cell memory. J Immunol.

[112] Walsh KB, Teijaro JR, Zuniga EI (2012). Toll-like receptor 7 is required for effective adaptive immune responses that prevent persistent virus infection. Cell Host Microbe.

[113] Clingan JM, Matloubian M (2013). B Cell-intrinsic TLR7 signaling is required for optimal B cell responses during chronic viral infection. J Immunol.

[114] Lam JH, Smith FL, Baumgarth N (2020). B cell activation and response regulation during viral infections. Viral Immunol.

[115] Upasani V, Rodenhuis-Zybert I, Cantaert T (2021). Antibody-independent functions of B cells during viral infections. PLoS Pathog.

[116] Roltgen K, Boyd SD (2021). Antibody and B cell responses to SARS-CoV-2 infection and vaccination. Cell Host Microbe.

[117] Burton AR, Maini MK (2021). Human antiviral B cell responses: emerging lessons from hepatitis B and COVID-19. Immunol Rev.

[118] Anwar MA, Shah M, Kim J, Choi S (2019). Recent clinical trends in Toll-like receptor targeting therapeutics. Med Res Rev.

[119] Ma Z, Zhang E, Gao S, Xiong Y, Lu M (2019). Toward a functional cure for hepatitis B: the rationale and challenges for therapeutic targeting of the B cell immune response. Front Immunol.

[120] Gray GE, Laher F, Lazarus E, Ensoli B, Corey L (2016). Approaches to preventative and therapeutic HIV vaccines. Curr Opin Virol.

[121] Trovato M, Sartorius R, D'Apice L, Manco R, De Berardinis P (2020). Viral emerging diseases: challenges in developing vaccination strategies. Front Immunol.

[122] Harbecke R, Cohen JI, Oxman MN (2021). Herpes zoster vaccines. J Infect Dis.

[123] Raven SFH, Hoebe C, Vossen A (2020). Serological response to three alternative series of hepatitis B revaccination (Fendrix, Twinrix, and HBVaxPro-40) in healthy non-responders: a multicentre, open-label, randomised, controlled, superiority trial. Lancet Infect Dis.

[124] Rosalik K, Tarney C, Han J (2021). Human papilloma virus vaccination. Viruses.

[125] Hong S, Zhang Z, Liu H (2018). B cells are the dominant antigen-presenting cells that activate naive CD4(+) T cells upon immunization with a virus-derived nanoparticle antigen. Immunity.

[126] Guo C, Peng Y, Lin L (2021). A pathogen-like antigen-based vaccine confers immune protection against SARS-CoV-2 in non-human primates. Cell Rep Med.

[127] Prates-Syed WA, Chaves LCS, Crema KP (2021). VLP-based COVID-19 vaccines: an adaptable technology against the threat of new variants. Vaccines (Basel).

[128] Kim C, Kim JD, Seo SU (2022). Nanoparticle and virus-like particle vaccine approaches against SARS-CoV-2. J Microbiol.

[129] Hemmati F, Hemmati-Dinarvand M, Karimzade M (2022). Plant-derived VLP: a worthy platform to produce vaccine against SARS-CoV-2. Biotechnol Lett.

[130] Reilley MJ, Morrow B, Ager CR (2019). TLR9 activation cooperates with T cell checkpoint blockade to regress poorly immunogenic melanoma. J Immunother Cancer.

[131] Kim H, Khanna V, Kucaba TA (2019). Combination of sunitinib and PD-L1 blockade enhances anticancer efficacy of TLR7/8 agonist-based nanovaccine. Mol Pharm.

[132] Mullins SR, Vasilakos JP, Deschler K (2019). Intratumoral immunotherapy with TLR7/8 agonist MEDI9197 modulates the tumor microenvironment leading to enhanced activity when combined with other immunotherapies. J Immunother Cancer.

[133] Bertoletti A, Tan AT (2020). HBV as a target for CAR or TCR-T cell therapy. Curr Opin Immunol.

[134] Meng F, Zhao J, Tan AT (2021). Immunotherapy of HBV-related advanced hepatocellular carcinoma with short-term HBV-specific TCR expressed T cells: results of dose escalation, phase I trial. Hepatol Int.

[135] Weinkove R, George P, Dasyam N, McLellan AD (2019). Selecting costimulatory domains for chimeric antigen receptors: functional and clinical considerations. Clin Transl Immunol.

[136] Kaczanowska S, Joseph AM, Guo J (2017). A synthetic CD8α:MyD88 coreceptor enhances CD8(+) T-cell responses to weakly immunogenic and lowly expressed tumor antigens. Cancer Res.

[137] Starck L, Popp K, Pircher H, Uckert W (2014). Immunotherapy with TCR-redirected T cells: comparison of TCR-transduced and TCR-engineered hematopoietic stem cell-derived T cells. J Immunol.

[138] Boni C, Barili V, Acerbi G (2019). HBV immune-therapy: from molecular mechanisms to clinical applications. Int J Mol Sci.

